# Current foveal inspection and previous peripheral preview influence subsequent eye movement decisions

**DOI:** 10.1016/j.isci.2022.104922

**Published:** 2022-08-13

**Authors:** Christian Wolf, Artem V. Belopolsky, Markus Lappe

**Affiliations:** 1 Institute for Psychology, University of Münster, Münster, Germany; 2 Department of Experimental and Applied Psychology, Vrije Universiteit Amsterdam, Amsterdam, the Netherlands

**Keywords:** Biological sciences, Neuroscience, Cognitive neuroscience

## Abstract

Humans visually inspect the world with their fovea and select new parts of the scene using saccadic eye movements. Foveal inspection and the decision of where and when to look next proceed simultaneously, but there is mixed evidence concerning their independence. Here, we tested their interdependence using drift-diffusion modeling. Participants first made a saccade to a predetermined inspection target and subsequently decided between two selection targets. We found that the inspected target’s meaningfulness and the opportunity to preview it peripherally affects fixation durations and the upcoming saccadic selection. Drift-diffusion modeling showed that meaningfulness and the absence of peripheral preview can both delay the subsequent saccadic decision process and affect the rate at which peripheral information is accumulated. Our results thus show that foveal inspection and peripheral selection are dependent on each other and that peripheral information can be maintained across the saccade to influence subsequent eye movement decisions.

## Highlights

• Foveally inspected targets affect the decision between targets in the periphery
• Meaningful foveal targets delay decision onset and affect decision outcome
• Prior peripheral preview of inspected targets modulated effects on decision outcome

## Graphical abstract

**Figure:**
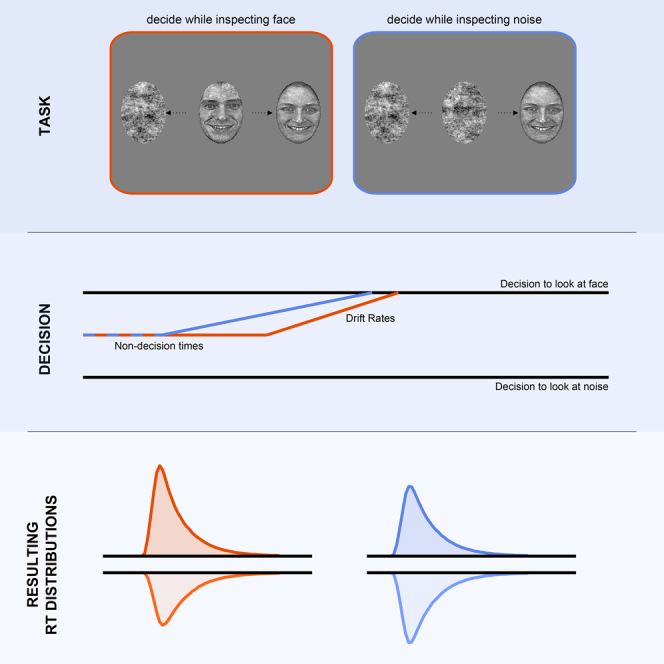

## Introduction

We use the central part of our visual field, the fovea, to inspect fine visual details, and we use our peripheral vision to select objects or regions of interest for future inspection. This differentiation is a consequence of the heterogeneity of visual acuity across the visual field (for review see Strasburger et al. (2011)). Visual acuity is highest in the fovea and declines in the periphery with increasing eccentricity. Consequently, we use peripheral vision to select objects of interest and inspect them one after the other using our fovea. Shifting our line of sight and thus our fovea is achieved by saccade eye movements. The visual sampling process is mostly restricted to periods of fixation, the time between two eye movements, because visual sensitivity is reduced during saccades (Zuber and Stark, 1966; Matin, 1974). Thus, every visual fixation comprises two processes: foveal inspection and peripheral selection. Processing priorities are not fixed within a fixation but follow the temporal dynamics that are expected from a visual system that uses eye movements to gather visual information. Perceptual performance in an attentional task was found to be initially highest for the foveally inspected target, whereas performance shortly before the onset of the next saccade is best at the future gaze position in the periphery (Rolfs et al., 2011). This is consistent with the idea of presaccadic shifts of visual attention that serve to enhance processing at the future saccade target (Kowler et al., 1995; Deubel and Schneider, 1996). Yet, whether the foveal inspection and peripheral selection are independent of each other or whether what we see influences the decision where to look next is not yet understood. And although the two processes typically co-occur during every fixation, most research on peripheral selection has neglected the possible influence of foveal content.

Ludwig et al. (2014) investigated whether foveal inspection and peripheral selection are interdependent or independent and whether the two operate in parallel. Therefore, they measured foveal inspection and peripheral selection simultaneously by constantly adding noise to a foveal inspection (tilt judgment) and a peripheral selection task (selection based on luminance contrast). This noise-classification approach allowed to reconstruct the time courses of both processes. Foveal inspection and peripheral selection were shown to proceed in parallel (Ludwig et al., 2014). Increasing the foveal task difficulty by reducing the tilt of the foveal target did not affect the peripheral selection process. This was taken as evidence that foveal inspection and peripheral selection operate independently (Ludwig et al., 2014). Likewise, increasing the processing difficulty of the foveal target by removing spatial frequencies does not affect peripheral target detection (Cajar et al., 2016b). Other findings suggest an interdependence of foveal inspection and peripheral selection. Physiologically, competition between fixation and saccade activity can be found throughout the oculomotor circuit (Munoz and Wurtz, 1993; Hanes et al., 1998). For example, fixation cells in superior colliculus start to reduce their activity before saccade onset (Munoz and Wurtz, 1993). Computationally, this competition between fixation and saccade activity is a crucial component of successful models on saccade target selection (Findlay and Walker, 1999; Tatler et al., 2017) and saccade timing (Engbert et al., 2005; Nuthmann et al., 2010; Laubrock et al., 2013). Behaviorally, fixation durations depend not only on the availability of visual information in the fovea but also on the availability of peripheral information (i.e., low spatial frequencies; Laubrock et al., 2013; Cajar et al., 2016a; Cajar et al., 2016b; Einhäuser et al., 2020). For example, Laubrock et al. (2013) measured fixation durations when a high-pass or low-pass filter was applied to either the periphery or the fovea. They observed that fixation durations were longer when the relevant information for each part of the visual field remained intact, thus when high spatial frequency information was available from the fovea and when low spatial frequency information was available from the periphery. They were able to reproduce fixation durations using a random-walk model that assumes that peripheral and foveal processing evolve in parallel and that high foveal processing demands can delay the next saccade. However, the fact that fixation durations covary with foveal and peripheral scene content does not necessarily imply that foveal inspection and peripheral selection interact. A prolonged foveal inspection could, for example, delay the onset of the selection process without affecting the selection process itself. Thus, these previous approaches cannot exclude the possibility that foveal and peripheral information processing merely covary but do not interact with each other. However, if foveal inspection not only affects when the next eye movement is made but also which peripheral target is selected for future inspection, then this could be taken as evidence that foveal inspection and peripheral selection interact.

**Figure 1:**
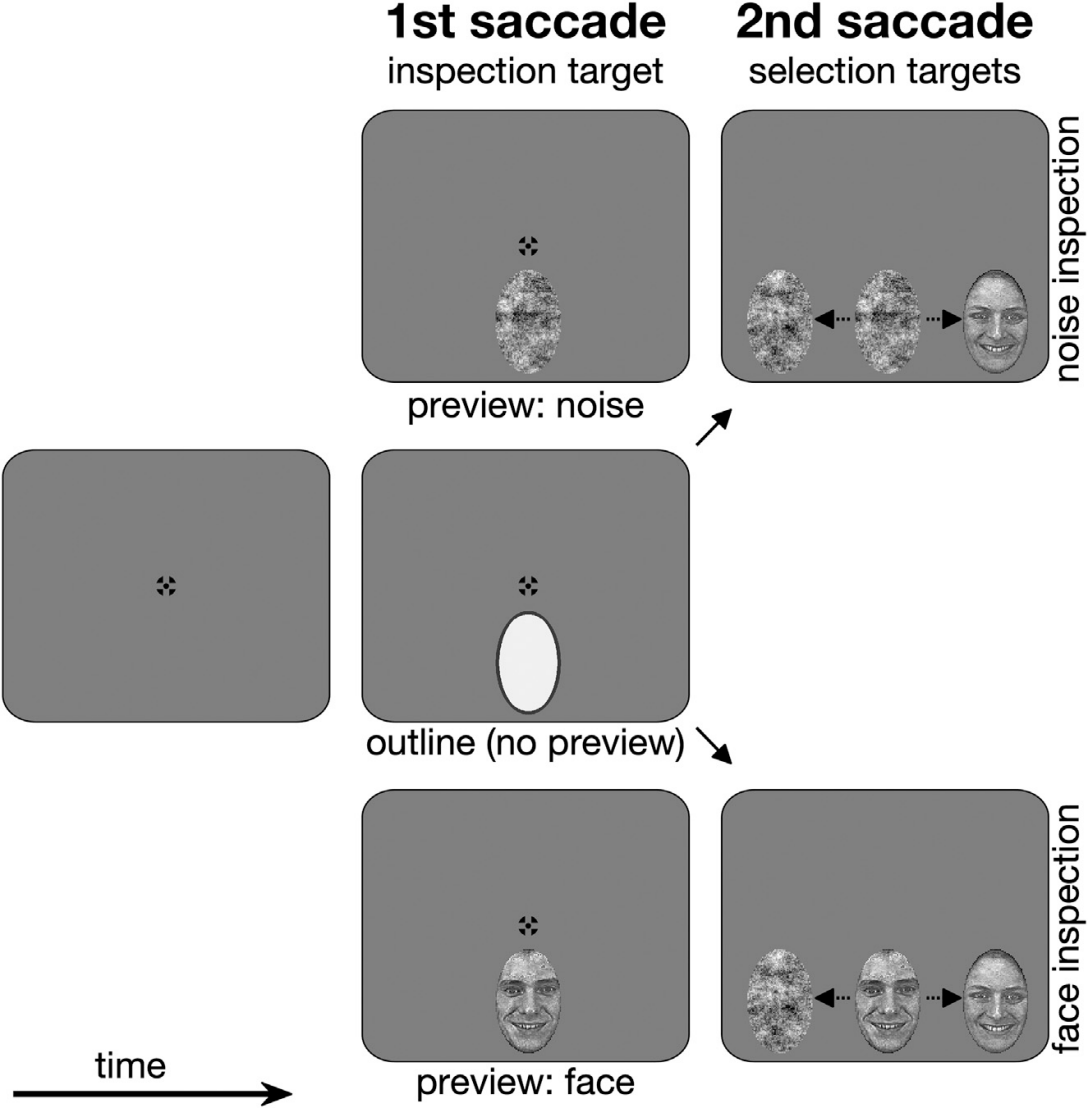
Trial procedure Trials started if gaze was detected on the central black fixation cross for 150 ms (left panel). After a uniform random interval, the first saccade target (the inspection target) appeared either above or below the current gaze position (center column). This inspection target could either be previewed in the periphery (top and bottom panels) or only its outline was shown (center panel). As soon as gaze was detected on the inspection target, the two selection targets were shown (and the identity of the inspection target was revealed in the outline condition; right column). Once participants looked at either of the two selection targets, all targets were removed from the screen after additional 500 ms. Stimuli are not drawn to scale. Examples for face stimuli (AF01HAS, AM29HAS) are taken from the Karolinska-directed emotional faces database (Lundqvist et al., 1998).

**Figure 2:**
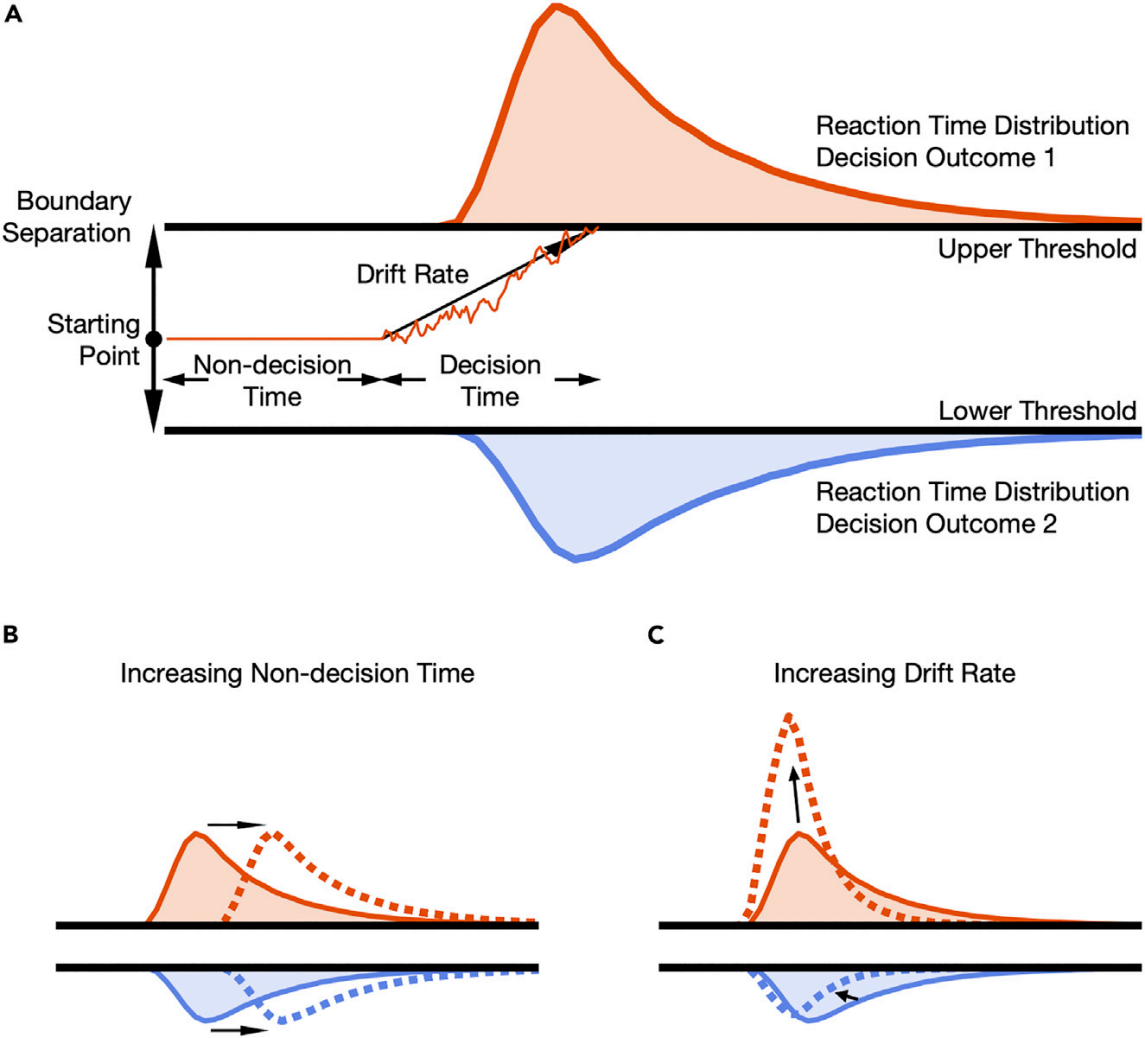
The drift-diffusion model and its parameters (A) The drift-diffusion model reflects noisy information uptake over time. It assumes that a response or decision is made once the accumulation process reaches either of two boundaries. Each boundary is associated with a different decision outcome, in our case making a saccade to the face versus to the noise patch. The drift process is composed of a random and a systematic component. The random component is reflected by noise in the model, whereas the systematic component is reflected by the drift rate, which denotes the mean evidence uptake per time. Typically, positive drift rates indicate an approach to the upper boundary and negative values an approach to the lower boundary. The thin orange line denotes an example trial, and the colored areas denote reaction time distribution for trials in which the upper (orange) or lower (blue) threshold was reached first. The four main parameters of the drift-diffusion model are the boundary separation, starting point, drift rate, and non-decision time. The model is often complemented with variability components of the latter three parameters. The boundary separation determines how much information is needed and can be affected by emphasizing either cautiousness or speed. The evidence accumulation signal origins in between the two boundaries, and the starting point can be affected by *a priori* biases toward either response alternative. The non-decision time reflects processes other than the decision itself, for example, perceptual or motor processes such as stimulus encoding or response execution. The drift rate can be affected by stimulus quality. Changes in the resulting reaction time distributions caused by an increase in non-decision time (B) or an increase in drift rate (C). Whereas changes in non-decision time only affect the reaction time, but not the decision outcome, changes in drift rate manifest itself in both variables: reaction time and decision outcome.

The aim of the present study was two fold. The first goal was to answer the question whether foveal inspection and peripheral selection interact. Therefore, we here measured peripheral selection in a dual saccade paradigm (Figure 1) and employed a drift-diffusion computational modeling approach to uncover whether foveal inspection and peripheral selection interact. The drift diffusion model (Ratcliff, 1978; for review see Ratcliff et al., 2016) can help to reveal latent decision processes based on the joint modeling of decision outcome and reaction time distributions (here: distribution of fixation fixations). It reflects noisy information uptake over time (Figure 2) and assumes that a response or decision is made once the accumulation process reaches either of two boundaries. In our paradigm (Figure 1), participants had to make two saccades on every trial. The first saccade target was predetermined (the inspection target). For the second saccade, participants had to decide between two alternative targets (the selection targets). Importantly, rather than introducing a foveal task and manipulating the difficulty of the foveal target (Ludwig et al., 2014), we manipulated target semantics, because eye movement control and foveal inspection are known to depend on cognitive processing demands and whether there is ample or little to see (Inhoff and Rayner, 1986; Kliegl et al., 2004; Einhäuser et al., 2020; Wolf & Lappe, 2021a, 2021b). Therefore, our targets were either meaningful face stimuli or meaningless noise patches.

In drift-diffusion modeling, each of the two boundaries is associated with a different decision outcome, in our case making a saccade to the face versus to the noise patch. The time dedicated to other processes than the decision is reflected in the non-decision time. Changing the non-decision time would affect the reaction time without affecting the decision outcome (Figure 2B). The drift rate on the other hand is the systematic component of the drift process and denotes the mean evidence uptake per time. Changes in the drift rate would affect both the decision outcome and the fixation duration (Figure 2C). Thus, if foveal inspection affects the peripheral selection process, we expect that foveal inspection would jointly affect fixation durations on the inspection target as well as the outcome of the decision between the two selection targets. Importantly, this should not only influence the nondecisional part of the selection process (non-decision time parameter in drift-diffusion model) but should also directly interfere with the decision itself (drift rate parameter, see Figure 2).

The second goal of the present study was to show whether the potential influence of foveal targets on peripheral selection is restricted to information obtained during the current fixation or whether information from previous fixations is combined for this purpose. To this end, we manipulated the peripheral preview of theinspection target before it was foveated. Having a peripheral preview or having no peripheral preview of the inspection target should not affect the decision between the selection targets if the foveal influence on peripheral selection is restricted to the current fixation. If, however, peripheral preview from the previous fixation modulates the influence of foveal inspection on peripheral selection, then this can be taken as evidence that peripheral information is maintained across the saccade and used for subsequent eye movement decisions.

## Results

To answer the question whether foveal inspection and peripheral selection interact, we measured peripheral selection in a free choice paradigm while manipulating which target participants were inspecting with their fovea. Targets were either meaningful face stimuli or meaningless noise patches that were matched for their low-level properties (Willenbockel et al., 2010). Thus, each decision was a decision between a peripheral face and a peripheral noise patch while inspecting a target of one of these two categories with the fovea (Figure 1). To answer the question whether peripheral information is maintained across the saccade and whether it can affect the subsequent selection process, participants first acquired the foveal inspection target by means of a saccade and could either preview the inspection target or only saw the stimulus outline. In the latter case, the outline was replaced with the inspection target as soon as gaze was detected on the inspection target. We modeled the selection outcome of the decision and the fixation duration on the foveal inspection target using drift-diffusion modeling (Figure 2). This allows us to distinguish whether changes in behavior can be attributed to the onset of the decision process or to changes of the decision process itself.

### Fixation durations and selection outcome

**Figure 3:**
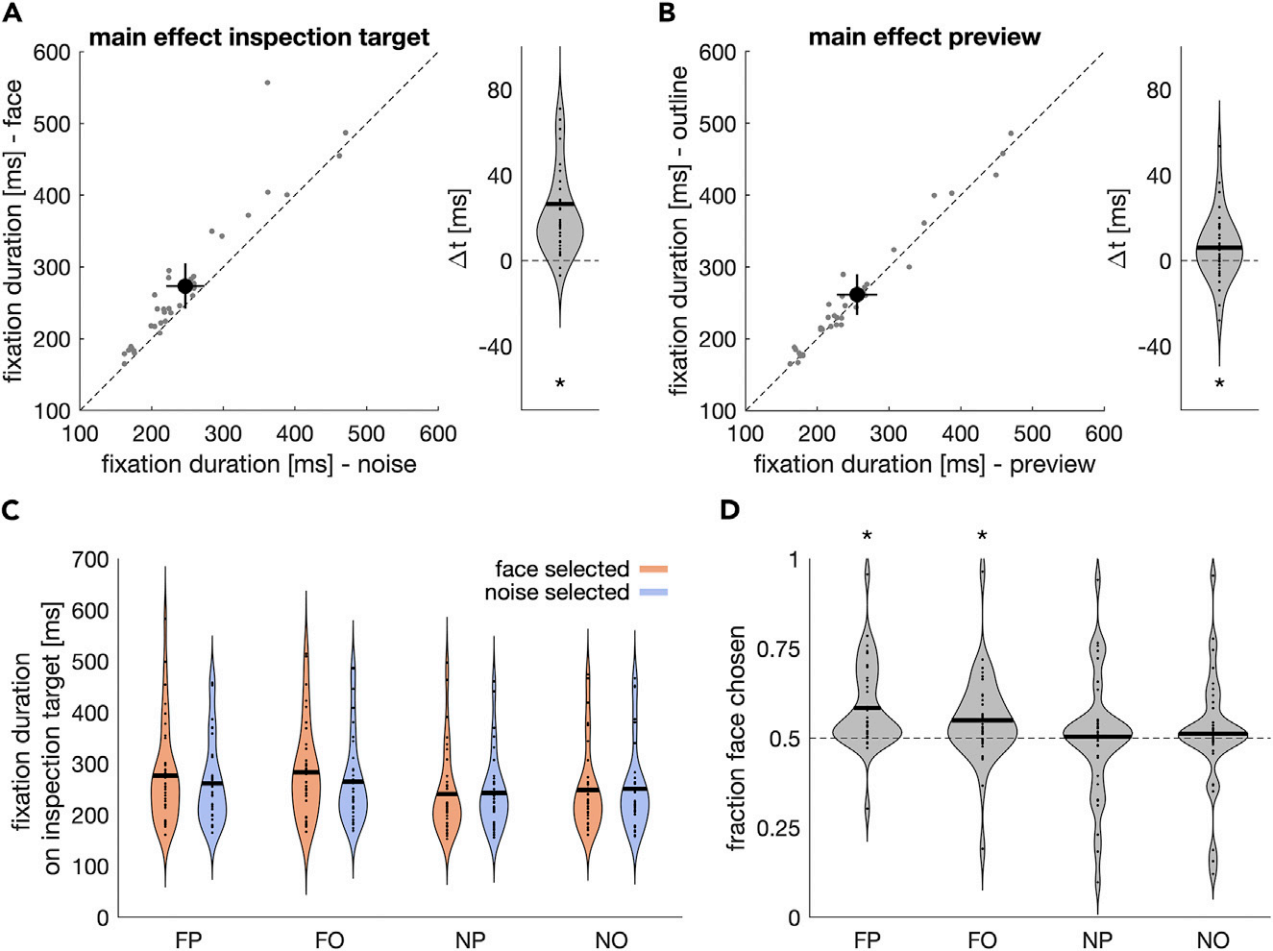
Fixation durations and selection outcome (A) Fixation durations on face and noise stimuli. Small gray dots denote the mean of individual participants; the black dot denotes the group mean with 95% confidence intervals of between-participant variability. The righthand panel shows a violin plot of the difference between the two, with positive values indicating longer fixation durations on face stimuli. The solid black line denotes the mean and the asterisk a significant difference (p < 0.05; t test). (B) Mean fixation durations on non-previewed (outline) and previewed (face or noise patch) stimuli. Same conventions as in A. (C) Violin plot of the mean fixation durations as a function of inspection target (F versus N), preview (P versus O), and decision outcome. (D) Violin plots of the proportion the face was chosen as selection target. Acronyms refer to the inspection target identity and whether it could be previewed (FP: face/preview, FO: face/outline, NP: noise/preview, NO: noise/outline). Asterisks denote a significant difference from 0.5 (p < 0.05; Wilcoxon-signed rank test).

Fixation durations on the inspection target showed large interindividual differences (Figure 3), which is expected because the task did not constrain reaction time. Yet, they varied systematically within individuals with the identity of the inspection target, *F*(1,35) = 30.83, p < 0.001, and the preview condition, *F*(1,35) = 6.14, p = 0.018. Fixation durations were higher when participants inspected a face (Figure 3A), *t*(35) = 5.55, p < 0.001, and when it had not been possible to preview the identity of the inspection target in the periphery (outline condition; Figure 3B), *t*(35) = 2.48, p = 0.018. In addition, fixation durations on the inspection target differed depending on the selection outcome, *F*(1, 35) = 12.44, p < 0.001. Fixation durations were higher in trials in which the face was selected for the secondary saccade (Figure 3C), *t*(35) = 3.53, p = 0.001, but mainly when the inspection target was itself a face, *F*(1, 35) = 19.44, p < 0.001 (inspection identity × selection outcome interaction). We observed a higher number of smaller saccades on the inspection target if the inspection target was a face (Figure S5).

Selection outcome also depended on the inspection target, *F*(1,35) = 8.19, p = 0.007, and on the peripheral preview, *F*(1,35) = 4.4, p = 0.043. When the inspection target was a noise patch, the selection outcome was on average balanced, yet prone to large interindividual differences (Figure 3D). When the inspection target was a face, participants more frequently decided to look at the face stimulus with the second saccade compared with when it was a noise patch, *t*(35) = 2.86, p = 0.007. Likewise, the face was more frequently chosen with the second saccade if the inspection target could have been previewed, *t*(35) = 2.10, p = 0.043. This was mainly the case when the inspection target was a face, *F*(1,35) = 16.75, p < 0.001 (identity × preview interaction): The difference between preview and no preview was stronger when the inspection target was a face compared with when it was a noise patch, *t*(35) = 4.09, p < 0.001.

### Foveal inspection changes selection time courses

Visual selection is known to unfold over time and its output may thus depend on whether an eye movement is initiated early or late (e.g., Schütz et al., 2012; Wolf and Lappe, 2020). We therefore wondered how foveal inspection of a meaningful versus meaningless target, and the peripheral preview thereof, affect the time course of oculomotor selection. We analyzed the time courses of the selection process as a function of the inspection target using smoothed response time courses and cluster-based permutation testing (SMART method; van Leeuwen et al., 2019; see Quantification and statistical analysis). This method allows to reconstruct time courses with high temporal precision for data with one data point per trial (e.g., a decision and the corresponding fixation duration). In contrast to binning methods, it does not require to give away information about the exact timing. SMART first computes a smoothed time series for every participant and subsequently a weighted aggregated time course that considers the temporal response distribution of every individual. A time course is then compared against a baseline or against another time course using cluster-based permutation testing.

**Figure 4:**
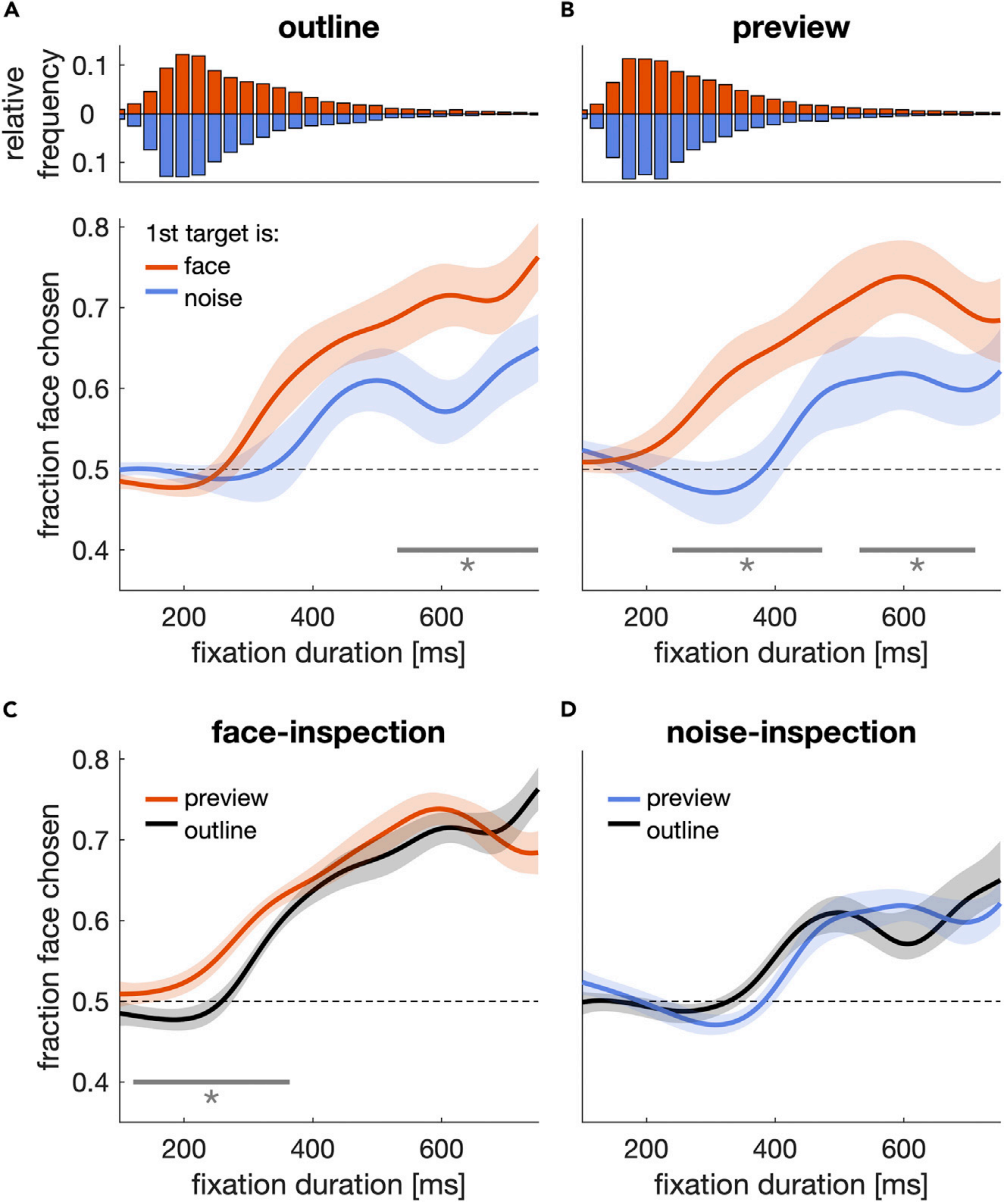
Selection over time (A and B) Fraction face chosen as a function of fixation duration for the outline (A) and preview (B) conditions. Orange (blue) color denotes a face (noise patch) as inspection target. Upper panels show fixation duration histograms of the respective conditions in the panel below. Lines indicate the mean, and shaded regions are 95% confidence intervals that result from comparing the two time courses (van Leeuwen et al., 2019). Solid horizontal lines and asterisks indicate a significant cluster (p < 0.05; SMART analysis). (C and D) Same data as in (A) and (B) but with a different combination of conditions.

Figures 4A and 4B show the aggregated time courses when the inspection target was a face or a noise patch, separate for the outline (Figure 4A) and the preview condition (Figure 4B). Face-inspection and noise-inspection time courses differed, both for the outline (p = 0.003, *t* = 617.37, *t*_*crit*_ = 355.62, 531–750 ms) and for the preview condition (p = 0.001, *t* = 753.67, *t*_*crit*_ = 376.50, 240–473 ms). Likewise, face-inspection time courses (Figure 4C) differed depending on peripheral preview of the inspection-target (p < 0.001, *t* = 815.60, *t*_*crit*_ = 363.41, 121–364 ms). No difference was observed between the two noise-inspection time courses (p = 0.064, *t* = 400.29, *t*_*crit*_ = 431.55, 286–436 ms) (Figure 4D). These time course differences suggest that foveal inspection and peripheral preview directly interfere with oculomotor selection. Although the preview effect is exclusive for faces (Figures 4C and 4D) and can affect selection comparatively early (>120 ms), the effect of foveal inspection emerges after longer fixation durations (>240 ms).

### Drift-diffusion modeling

**Figure 5:**
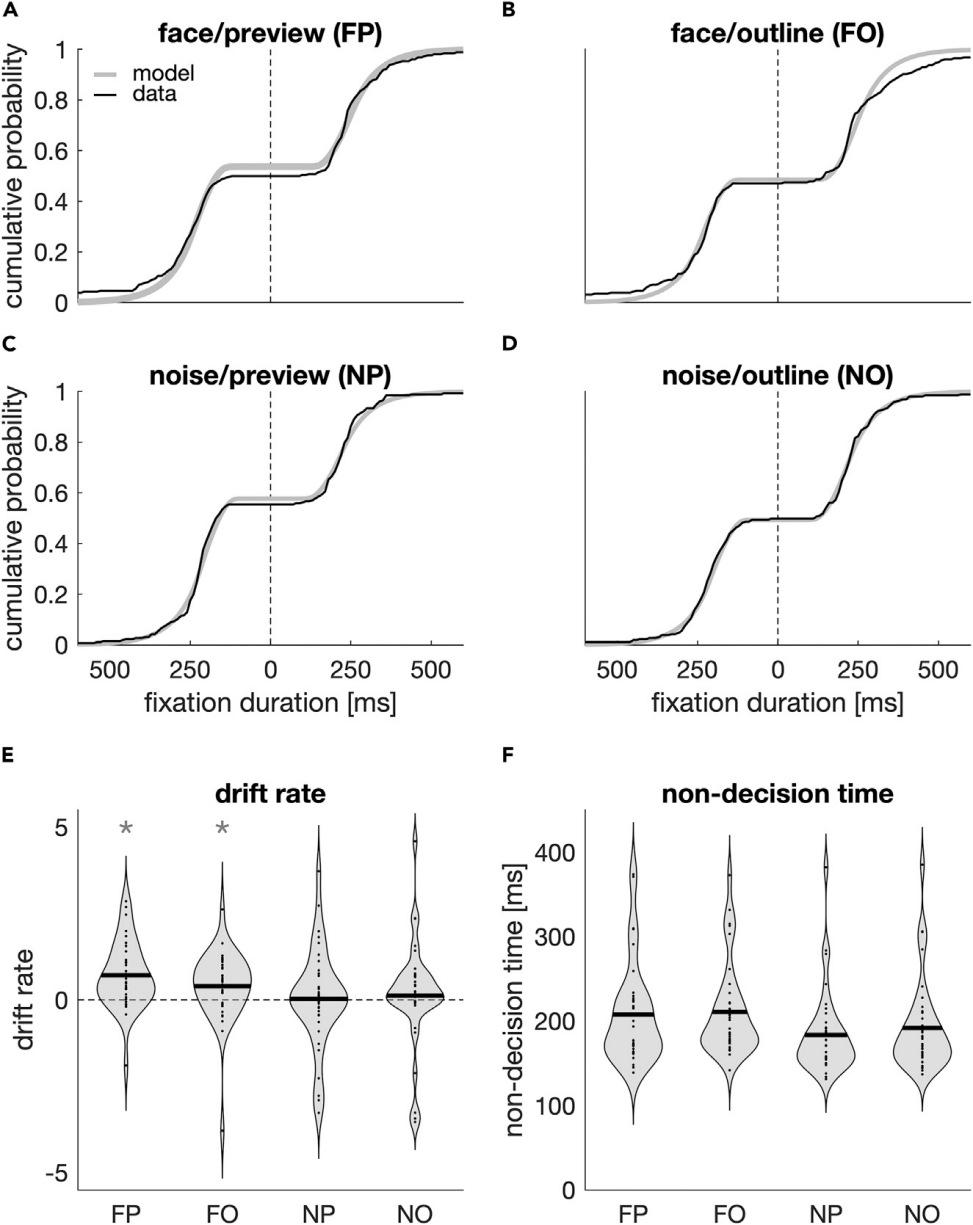
Drift-diffusion analysis (A–D) Cumulative density functions for one participant in the four respective conditions. Model predictions are depicted in gray, and the data are depicted in black. The left side of each panel (left from the dashed line) indicates responses to the noise patch. Thus, the intersection with the abscissa denotes the fraction of trials the noise patch was chosen. Please note that the left side of each graph (responses to the noise patch) is mirrored. (E and F) Violin plots for individual drift rate (E) and non-decision time (F). Dots are the parameters of individual participants; black lines denote the group mean. Non-decision times differ with respect to inspection target (F > N) and preview condition (O > P). Drift rates display an inspection target × preview interaction. Acronyms are the same as in Figure 3. Asterisks indicate a drift rate different from 0 (p < 0.05; t test). See also Figures S3–S5.

To further quantify how foveal inspection and peripheral preview affect oculomotor selection, we employ a drift-diffusion model approach (Ratcliff, 1978; for review see Ratcliff et al., 2016; Figure 2). Specifically, we were interested whether foveal inspection of a semantically meaningful versus a meaningless target, and the peripheral preview thereof, delayed the onset of the selection process or whether they interfered with the selection process itself (or both). To this end, we fit eight different versions of the drift-diffusion model to the data in which the drift rate, non-decision time, boundary separation, or any combination of the three parameters was allowed to vary as a function of inspection target and preview. The drift rate reflects the systematic component of the drift process and denotes the mean evidence uptake per time. Each reaction time is thought to be the result of this decisional drift process along with a non-decision time. This latter component reflects the part of the reaction time that is not devoted to the evidence accumulation process, but is, for example, devoted to motor or perceptual processes. The boundary separation determines how much information is needed for a decision and can be affected by emphasizing either cautiousness or speed. From these eight models, we selected the best-fitting model based on the highest information weights (Burnham and Anderson, 2004; see Quantification and statistical analysis) derived from the Bayesian information criterion (BIC). The model in which drift rate and non-decision time were allowed to vary across conditions provided the best fit to the data as reflected in the highest mean information weight (Figure S3). Figures 5A–5D show cumulative density functions for empirical data and model predictions for an exemplary observer.

We compared the derived drift-rate and non-decision time parameters using a 2 × 2 repeated-measures ANOVA with the factors inspection target (face vs noise) and preview (preview vs outline). Drift rates (Figure 5E) were highest in the face/preview condition (FP), M_FP_ = 0.607, SE_FP_ = 0.129; lowest in the noise/preview condition (NP), M_NP_ = −0.024, SE_NP_ = 0.198; and intermediate in the face/outline (FO), M_FO_ = 0.320, SE_FO_ = 0.131, and noise/outline (NO) condition, M_NO_ = 0.096, SE_NO_ = 0.215. Drift rates were different from zero, whenever a face was inspected (*t*_*FP*_ = 4.70, p < 0.001, *t*_*FO*_ = 2.45, p = 0.02) but not when the inspection target was noise patch (*t*_*NP*_ = 0.123, p = 0.903, *t*_*NO*_ = 0.445, p = 0.659). This pattern of results was reflected in the inspection target × preview interaction (*F*(1,35) = 12.80, p = 0.001). The difference in drift rates between face and noise inspection was larger for the preview compared with the outline condition (*t*(35) = 3.06, p = 0.004). We neither observed a main effect of inspection target (*F*(1,35) = 3.92, p = 0.056) nor a main effect of preview (*F*(1,35) = 1.80, p = 0.188).

Non-decision times are depicted in Figure 5F. Descriptively, nondecision times were highest in the face/outline condition (FO), M_FO_ = 211 ms, SE_FO_ = 18.9 ms; lowest in the noise/preview (NP) condition, M_NP_ = 183 ms, SE_NP_ = 17.0 ms; and in-between the two in the face/preview (FP), M_FP_ = 208 ms, SE_FP_ = 20.4 ms, and the noise/outline condition (NO), M_NO_ = 192 ms, SE_NO_ = 18.6 ms. The ANOVA on the non-decision time revealed a main effect of inspection target, *F*(1,35) = 29.02, p < 0.001. Non-decision times were higher when the inspection target was a face compared with when it was a noise patch, *t*(35) = 5.39, p < 0.001. This highlights the prolonged foveal inspection for semantically meaningful targets. Furthermore, non-decision times depended on the peripheral preview, *F*(1,35) = 7.30, p = 0.011, and were higher in the outline compared with the preview condition, *t*(35) = 2.70, p = 0.011, reflecting a peripheral preview benefit. We observed no inspection target × preview interaction, *F*(1,35) = 1.34, p = 0.254.

## Discussion

Our study shows that foveal inspection and peripheral selection are not independent processes and that the semantics of a foveally inspected target (whether it is meaningful or meaningless) directly interferes with the decision where to look next. Drift-diffusion modeling suggests that foveal inspection of a meaningful target interfered with peripheral selection in two ways: (1) it delayed the onset of the decision where to look next (as reflected in higher non-decision times) and (2) it increased the drift rates (Figure 5), resulting in a more frequent selection of meaningful targets when the inspection target was meaningful itself (Figures 3 and 4).

The present differences in drift rate and non-decision time further depended on whether the inspection target could be previewed with the periphery: peripheral preview decreased non-decision times and modulated the increase in drift rates. These reduced non-decision times can be considered a further example of the well-established peripheral preview benefit: better or faster processing of foveated objects that had been previewed in the periphery prior to the saccade (for review see Huber-Huber et al., 2021). Yet, we cannot ultimately distinguish whether fixation durations and non-decision times decreased due to the peripheral preview or whether they increased in the outline condition due to a trans-saccadic prediction error. Our visual system predicts foveal input based on the peripheral preview (Bompas & O’Regan, 2006; Herwig and Schneider, 2014; Bosco et al., 2015; Valsecchi and Gegenfurtner, 2016), which can explain the trans-saccadic benefit in perceptual performance with congruent information (Ganmor et al., 2015; Wolf and Schütz, 2015). Changing the target during the saccades (as in the outline condition) violates this prediction, resulting in a trans-saccadic prediction error. Prediction errors can be observed even when the trans-saccadic change goes unnoticed. In our case, the trans-saccadic change from the outline to the face or noise patch was highly conspicuous and might have caused a trans-saccadic prediction error that increased fixation durations. Distinguishing between a decrease due to preview or an increase due to a trans-saccadic prediction error would require an appropriate baseline or the modulation of the prediction error magnitude. However, we would expect that a trans-saccadic prediction error would affect face and noise images equally, whereas preview effects could be exclusive for faces, for example due to differences in target semantics (Yan et al., 2009; Schotter, 2013). Whereas we observed such an interaction between target identity and preview in selection outcome (Figure 3) and drift rates (Figure 5), it was not present in fixation durations or non-decision times. Even if our results cannot differentiate between an increase or decrease in fixation durations, the present drift-rate results further highlight the functional role of peripheral preview not only for efficient perception after the saccade but also for subsequent motor decisions.

Our results are inconsistent with the results reported by Ludwig et al. (2014) who found independence of foveal inspection and peripheral selection. These discrepancies might be explained by the different ways to manipulate foveal inspection. Ludwig et al. (2014) manipulated the difficulty of a foveal task by changing the mean orientation contrast, whereas our manipulation of target semantics can be considered more high-level processing. Although they found that foveal task difficulty did affect perceptual performance, it did not affect fixation durations. It thus can be speculated that their manipulation of perceptual task difficulty did not affect foveal inspection because either (1) the amount of information that could be extracted from a difficult and easy target was the same (i.e., its orientation), (2) their foveal task required aggregation of information across multiple successively shown gratings, or (3) because of the time constraints introduced by their experiment.

In line with findings from the reading literature (e.g., Kliegl et al., 2004; Payne et al., 2016), our present results show that foveal inspection is susceptible to target semantics and thus to the possibility to extract visual information. Yet, we cannot distinguish whether the discrepant results are caused by the fact that target semantics are more successful in affecting foveal load than manipulations of task difficulty, by the different level of processing (high-level versus low-level) or simply by the fact that our task was temporally unconstrained. Furthermore, our task did not require a deeper foveal analysis of the inspection target. This is different from Ludwig et al. (2014) and many natural tasks in which analyzing the foveal target is crucial for perceptual performance. Yet, the systematic differences between face and noise patch show that the inspection targets were not fully ignored. Moreover, inspection of faces and noise patches differed in terms of the number of smaller saccades that were made to explore the inspection target (Figure S2). Thus, foveal targets, particularly faces, were not neglected but analyzed, even in the absence of a foveal task. Introducing a foveal task might have rendered the otherwise less relevant noise patches equally relevant as the inherently relevant faces, considering that perceptual tasks and meaningful images give rise to a comparable oculomotor signature (Wolf and Lappe, 2021b). Hence, one possible explanation why the drift rate was exclusively affected by faces could be that meaningful images (e.g., faces) are more likely to be processed without a task (Devue et al., 2012), whereas meaningless and irrelevant stimuli (e.g., noise patches) are only equivalently analyzed once they are rendered relevant by the existence of a perceptual task.

When selecting between differently valued options (food items) and given the possibility to switch gaze back and forth, people tend to select that item that they previously fixated for a longer duration (Krajbich et al., 2010; Krajbich and Rangel, 2011). These results were captured by a modified version of the drift-diffusion model (attentional drift-diffusion model, aDDM), which makes the additional assumption that a visual fixation affects the drift rate of the decision process in favor of the fixated option (Krajbich et al., 2010). Yet, alternative explanations have been suggested for the underlying empirical relationship between overt attention and selection behavior, for example effort-avoidance and striving for consistency (Mormann and Russo, 2021). Our present results show that visual inspection can affect drift rate and thus choice behavior within a single fixation. Moreover, peripheral preview of a not-yet-inspected target can enhance this effect. Unlike in the experiments by Krajbich and colleagues, in our paradigm inspected target and the choice options were not identical but belonged to the same category. Relating the core principle of aDDM to our findings, one might suspect that the effect of inspection target and preview on the drift rate was exclusive for faces, because faces were inspected longer than noise patches (Figure 3). Consequently, there would have been more time for the inspected face to affect the drift process. Our results refute this assumption: if differences in decision outcome and drift rate were caused by the difference in fixation duration, we would have expected that the selection time courses (Figure 4) were the same and that choices were increasingly biased toward the noise patch (face) the longer another noise patch (face) was inspected. Instead, the second saccade favored the face the longer any of the inspection targets were fixated (Figure 4).

The aDDM describes how decisions are affected by overt attention (i.e., visual fixations). Yet, our drift rate results might reflect differences in covert attention and the dynamics of attention and priority within a fixation. At the beginning of a fixation, priority is highest at the fovea (Rolfs et al., 2011), whereas processing priority is highest at the future saccade goal shortly before the next saccade is made (Rolfs et al., 2011; Deubel and Schneider, 1996; Kowler et al., 1995). Which target is selected as upcoming saccade target is often explained in terms of a priority map, a two-dimensional representation of space, in which the highest activity within the map determines the upcoming saccade target. The activity (i.e., priority) is determined by the combination of the targets’ salience and their behavioral relevance (for reviews see Fecteau and Munoz, 2006; Bisley and Goldberg, 2010; Belopolsky, 2015). Characteristics of such a map can be found in many neural sites within the oculomotor system that also show characteristics of evidence accumulation as it is reflected in the drift-diffusion model (for review see Schall, 2019). Yet, our understanding of the mapping between accumulator models like the drift diffusion model on the one hand and neural activity or cognitive processes like attention on the other hand is still far from being complete (Schall, 2019). Whereas covert attention certainly plays a role in peripheral selection and may contribute to differences between conditions, we here view it as an integral part of the evidence accumulation process guiding the saccadic response.

To sum up, we here show that inspecting a target in the fovea can affect peripheral target selection mechanisms and thus where we subsequently move our eyes. Our results support the view that foveal and peripheral processing are not independent and are thus in line with many successful models of saccade generation (e.g., Findlay and Walker, 1999; Engbert et al., 2005; Nuthmann et al., 2010; Laubrock et al., 2013; Tatler et al., 2017) that emphasize the competition of foveal and peripheral processing for saccade target selection. Furthermore, we show the influence of a foveal target on saccade target selection is not restricted to information gathered within a single fixation. Instead, it can be modulated by information gathered from the previous fixation, that is, when the foveal target had been previewed in the periphery.

### Limitations of the study

The generalization of our results might be restricted to situations in which the foveal inspection target shares features with any possible eye movement target: foveal inspection of a certain target category (e.g., a face) may prime the corresponding action (e.g., selecting a face as upcoming saccade target), which, in turn, would affect the outcome of the peripheral selection process. Such a priming-based mechanism would be related to the ideomotor principle (James, 1890; for review see Shin et al., 2010) and explain why the drift rate findings are exclusive for faces. According to this principle, actions are represented by their anticipated effects, and these action effects have been shown to contribute to oculomotor control (e.g., Huestegge and Kreutzfeldt, 2012; Pfeuffer et al., 2016). If that were the case, then our findings might only be true in situations in which foveal and peripheral targets belong to the same category or at least share perceptual features. This possible caveat remains to be tested by future studies.

The evidence in favor of the model where drift rate and non-decision time were allowed to vary was not fully unambiguous (Figure S3). Therefore, to reveal the robustness of our results, we compared drift rate and non-decision time parameters with the parameters that were obtained when boundary separation was additionally allowed to vary across conditions. This is particularly important because changes in boundary separation can be captured as changes in non-decision time when fitting the drift-diffusion model (Dutilh et al., 2019). Whereas the statistical pattern of drift rates was not affected by the additional degrees of freedom of the boundary separation, one of the two main effects observed in non-decision time was now observed in the boundary separation (Figure S3). When the boundary separation parameter was additionally allowed to vary across conditions, non-decision times were still higher when the inspection target was a face, yet the main effect of preview was instead observed in the boundary separation parameter, with a higher boundary separation in the outline compared with the preview condition. Please note that the model without boundary separation provided a better fit to the data as judged by information weights. Yet, the finding that peripheral preview affected non-decision times should be interpreted carefully.

## STAR★Methods

### Key resources table

**Table:** 

REAGENT or RESOURCE	SOURCE	IDENTIFIER
**Deposited data**		

Eye movement and perceptual data	Zenodo.org	10.5281/zenodo.6323227

**Software and algorithms**		

MATLAB R2021a	Mathworks, Natick, MA, USA	https://www.mathworks.com/products/matlab.html, RRID:SCR_001622
Psychtoolbox 3, including Eyelinktoolbox	Brainard (1997); Kleiner et al. (2007); Cornelissen et al. (2002)	http://psychtoolbox.org/, RRID:SCR_002881
JASP (Version 0.10.2)	Wagenmakers et al., (2018)	https://jasp-stats.org/, RRID:SCR_015823

**Other**		

Eyelink 1000 + eye tracker	SR Research Ltd., ON, Canada	https://www.sr-research.com/products/eyelink-1000-plus/, RRID:SCR_009602

### Resource availability

#### Lead contact

Further information and requests for resources should be directed to and will be fulfilled by the lead contact, Christian Wolf (chr.wolf@wwu.de).

#### Materials availability

This study did not generate new unique reagents.

### Experimental model and subject details

We recorded data of 36 individuals (median age: 21, age range: 18–30, 7 males, 29 females, 7 males). Participants were undergraduate students from the University of Münster and received course credit or 8€/h for participation. Written informed consent was provided before testing. The experiment was approved by the ethics committee of the Department of Psychology and Sport Sciences of the University of Münster and conducted in accordance with the declaration of Helsinki.

### Method details

#### Setup

Stimuli were presented on an Eizo FlexScan 22-inch CRT monitor (Eizo, Hakusan, Japan) with a resolution of 1152 × 870 pixels, a refresh rate of 75 Hz, and an effective display size of 40.7 × 30.5 cm. Participants viewed the monitor from a 67 cm distance. We controlled stimulus presentation via the Psychtoolbox (Brainard, 1997; Kleiner et al., 2007) in MATLAB (The MathWorks, Natick, MA) and recorded eye position of the right eye at 1000 Hz using the EyeLink 1000 (SR Research, Mississauga, ON) and the EyeLink Toolbox (Cornelissen et al., 2002). All stimuli were presented on a uniform gray background.

#### Stimuli

Targets were either faces or noise patches. These two classes of stimuli have been successfully used to study how meaningful targets affect eye movement behavior (Xu-Wilson et al., 2009; Meermeier et al., 2016). We used 128 face stimuli, taken from different databases, 70 from the Karolinska directed emotional faces database (KDEF; Lundqvist et al., 1998), 32 from the dataset collected at the European Conference on Visual Perception (ECVP) in Utrecht (http://pics.stir.ac.uk/), 10 from the CVL database provided by the Computer Vision Laboratory, University of Ljubljana, Slovenia (Peer et al., 2014), 16 from the Multi-Racial Mega-Resolution database (MR2; Strohminger et al., 2016). Images from each database contained the same amount of male and female faces, and all faces were shown from a frontal view with smiling or neutral expression. Images were seen through an elliptic window with a height of 2.5° and width of 1.55°. We accounted for low-level information in the images by matching face and noise stimuli with respect to spatial frequency and luminance profile using the SHINE toolbox (Willenbockel et al., 2010). As outline, we used a dark elliptic ring with bright center (Figure 1). This stimulus and its properties were chosen because it elicited similar reactive saccade latencies as faces and noise patches in a pilot version of the experiment. Yet, in the actual experiment, latencies of the first saccade toward the elliptic outline were slightly increased (see Figure S1).

#### Procedure and design

Participants started each trial by looking at a central black fixation cross for 150 ms. A combination of bull’s eye and hair cross was used as fixation cross (Thaler et al., 2013). After a random delay between 300 and 700 ms, the first saccade target (the inspection target) appeared 8° above or below the fixation cross. As soon as gaze was detected on this inspection target (distance <2.5° between gaze and target center), the two selection targets appeared left and right at an eccentricity of 12°, constituting the potential targets for the second saccade. Selection targets always consisted of one face and one noise stimulus. When gaze was detected on a selection target, all targets were removed from the screen after additional 500 ms and the next trial could be started. Participants were instructed to look at two targets in each trial, the inspection target and one of the selection targets. No explicit instruction was provided about the selection behavior, for example choosing both target categories equally often and doing so in a random order. Whereas the inspection target was always from the same category as one of the selection targets, we never showed the exact same target within one trial.

The design comprised two within-participant variables with two levels each: The identity of the inspection target (face vs noise) and whether it could be previewed or not (preview vs outline). Combination of these two factors resulted in four experimental conditions: FP (face/preview), FO (face/outline), NP (noise/preview), NO (noise/outline). Participants performed 256 trials per condition, resulting in 1024 trials. Consequently, each stimulus was shown eight times as selection target and four times as inspection target. Trials from all conditions were randomly interleaved and a break was included after every 205 trials.

### Quantification and statistical analysis

We measured eye position of the right eye using the EyeLink 1000. Saccade onsets and offsets were defined offline using the EyeLink-1000 algorithm. To compare mean fixation durations, we used a 2 × 2 × 2 repeated-measures ANOVA with the factors inspection target (face vs noise), preview (preview vs outline) and selection outcome (face vs noise). The ANOVA was conducted in JASP (Version 0.10.2; Wagenmakers et al., 2018). We used Wilcoxon-signed rank tests to indicate whether selection outcome was different from chance (i.e., different from 0.5; Figure 2D) and t-tests to indicate whether drift rates (Figure 5E) and differences in fixations durations (Figures 2A and 2B) were different from 0.

To analyze the time course of selection outcome, we used the SMART procedure (smoothing method for the analysis of response time courses, van Leeuwen et al., 2019), where the data are (i) temporally smoothed for every individual, (ii) a weighted time-course is constructed that considers the distribution of data for every individual and (iii) a cluster-based permutation test is performed to compare two time courses or one time course against baseline. Data were analyzed at a 1 ms resolution. We smoothed the data with a Gaussian kernel of 48 ms width and used 1000 permutations for every test. Time courses were compared in a time window between 100 and 750 ms (fixation duration on inspection target). For every comparison we report four values: the p value, the cluster strength of the nonpermuted data (*t*), the 95^th^ percentile of the permuted distribution, and the time window of the (strongest) significant cluster. The 95^th^ percentile of the permuted cluster strength distribution is the critical *t* value (*t*_*crit*_) to which the cluster strength of the nonpermuted data is compared. The p value is given by the relative position (i.e., percentile) of the nonpermuted cluster strength in the distribution of all permuted cluster strengths.

We fit the drift-diffusion model (Figure 2) to the data using fast-dm-30 (Voss and Voss, 2007; Voss et al., 2015). The data was coded such that the upper threshold was associated with the decision to look at the face and the lower threshold with a decision to look at the noise patch. To select a model that fits the data best, we fit eight different versions of the drift diffusion model. All models included boundary separation, starting point, drift rate, non-decision time as well as intertrial variability of the latter three. However, drift rate, boundary separation, and the non-decision time, or any combination of the three were allowed to vary across condition. The starting point is typically affected by prior information, for example by a cue indicating a higher probability or a higher payoff associated with one of the two response options (e.g., Mulder et al., 2012). Given that the direction of stimuli was fully balanced, and participants had no prior information before the start of each trial, we did not include the starting point in the model selection process for reasons of parsimony. Model selection was based on information weights (Burnham and Anderson, 2004) derived from the Bayesian information criterion (BIC) for each of the eight different model versions. Information weights range from 0 to 1 and higher values indicate a better fit for the respective model. The model in which drift rate and non-decision time were allowed to vary provided the best fit to the data as indicated by the highest mean information weights (Figure S3).

The fast-dm toolbox assumes that the drift rate is normally distributed with mean *v* and SD *sv*, whereas the variability of starting point and non-decision time follow a uniform distribution with means *z* and *t0* and width *sz* and *st0* (Voss et al., 2015). Drift diffusion modeling, like modeling in general, provides many degrees of freedom to the researcher (Dutilh et al., 2019). Although differently set up models can converge in their results (Dutilh et al., 2019), we attempted to be reassured that our findings are robust to the different decisions that were made along the way. The Kolmogorov-Smirnov statistic was used for the optimization of parameters. This statistic is typically considered robust against outliers. When the model was fit with 5% of outliers removed, nearly the same parameters were reproduced, and we thus decided to use the full dataset of every individual (Figure S4). The maximum likelihood optimization criterion, in contrast to the Kolmogorov-Smirnov statistic, revealed more variable and partly implausible parameters (Figure S5). However, this can be caused by the fact that maximum likelihood is outlier sensitive. Yet, maximum likelihood parameters did not converge with an increasing number of outliers removed (Figure S5). We therefore considered the Kolmogorov-Smirnov statistic the best solution for our data. The algorithm for parameter estimation and optimization is described in Voss and Voss (2007).

## Data Availability

• Eye-movement data and perceptual data is deposited at Zenodo and will be publicly available as of the date of publication. The doi is listed in the key resources table. • This paper does not report original code. • Any additional information required to reanalyze the data reported in this paper is available from the lead contact upon request (Christian Wolf, chr.wolf@wwu.de).

## Supporting citations

Maris and Oostenveld (2007); Nystrom et al. (2017).
