# Supplementary material for: Current foveal inspection and previous peripheral preview influence subsequent eye movement decisions
Source: iScience. 2022 Aug 13;25(9):104922. doi: 10.1016/j.isci.2022.104922 (PMC9429799; doi:10.1016/j.isci.2022.104922)
Supplement: Document S1. Figures S1–S5 [file mmc1.pdf]

iScience, Volume *25*

Supplemental information

Current foveal inspection and previous peripheral preview influence subsequent eye movement decisions

Christian Wolf, Artem V. Belopolsky, and Markus Lappe

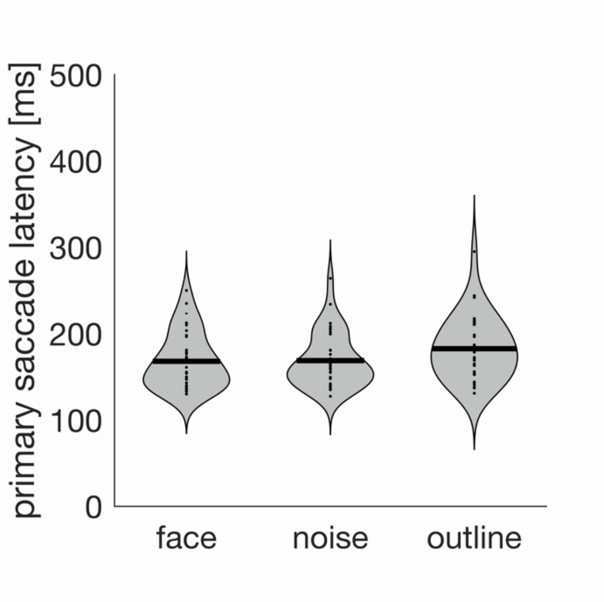

Supplementary Figure S1. Latencies of the first saccade to the inspection target, related to Figure 1 and Figure 3. Violin plot showing the aggregated mean (black line) and individual values (black dots) when the first saccade foveating the inspection target was made towards a face stimulus, a noise patch or towards the elliptic outline. Latencies were comparable for saccades towards the face, M_{face} = 168 ms, $CI_{95;face}$ = 22.0 ms, or noise patch, M_{noise} = 169, $CI_{95;noise}$ = 21.2 ms, and elevated for saccades towards the elliptic outline, $M_{outline}$ = 183 ms, $CI_{95;outline}$ = 24.2 ms.

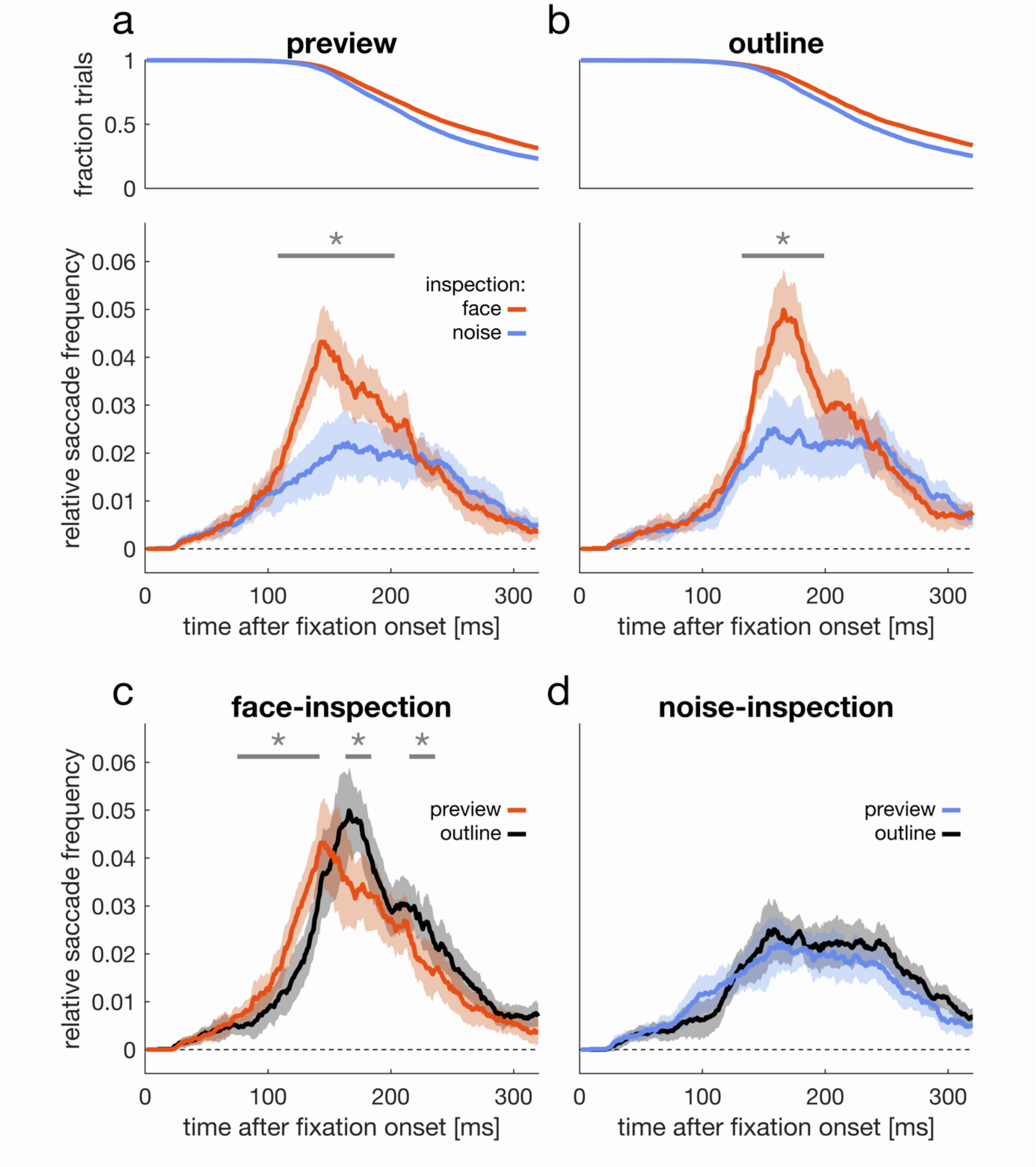

Supplementary Figure S2. Relative frequency of smaller saccades during inspection of the first target, related to Figure 3. (a, b) Lower panels show the fraction of trials with a saccadic frame when the inspection target was a face (orange) or a noise patch (blue) and when the inspection target could be previewed (a) or not (b). Shaded areas denote the 95% confidence interval of the difference between conditions. Upper panels show the descending cumulative probability of fixation durations for the respective time points shown below. (c, d) Same data as in (a) and (b) but with a different combination of conditions.

Fixation duration was defined as the time between the end of the saccade bringing gaze onto the inspection target and the start of the saccade bringing it away from it and towards either of the selection

targets. This definition allows that fixation durations include smaller intermediary saccades on the inspection target itself. These smaller saccades include microsaccades but also larger saccades that serve to explore the image. We compared the time courses of relative saccade frequency between face and noise inspection using a cluster-based permutation test (Maris & Oostenveld, 2007). For every timepoint while gaze was on the inspection target (i.e., millisecond) we computed the frequency of trials with a saccade relative to the total number of trials and compared time courses using 1000 permutations. The analysis was restricted to the 320 ms after fixation onset. Consistent with the SMART analysis from the main manuscript, we report the same four values for every comparison:

We observed a higher number of saccades when the inspection target was a face, compared to when it was a noise patch, both for the preview (panel a), $t = 460.79$, $t_{crit} = 48.23$, $p < 0.001$, 108–203 ms, as well as for the outline condition (panel b), $t = 351.76$, $t_{crit} = 50.11$, $p < 0.001$, 132–199 ms. We found no evidence for a difference between the preview and outline time course when the inspection target was a noise patch (panel d), $t = 54.14$, $t_{crit} = 54.34$, $p = 0.051$, 95–115 ms, but did so when the inspection target was a face (panel c), $t = 219.62$, $t_{crit} = 51.36$, $p < 0.001$, 75–142 ms. Relative saccade frequency for this strongest cluster was higher for the preview condition. Yet, later clusters showed the opposite effect.

Please note that we had recorded monocular eye movements. Detection of microsaccades typically relies on binocular recordings to reject monocular detections due to noise in the eye position signal (Nyström et al., 2017). Given that we only recorded eye position of one eye, we cannot exclude that some of the detected smaller saccades on the inspection target were caused by noise. This might render the absolute values of these smaller saccades less accurate. Yet, we have no reason to assume that this might have contributed to differences in relative saccade frequency between conditions, because the noise should be the same for all conditions.

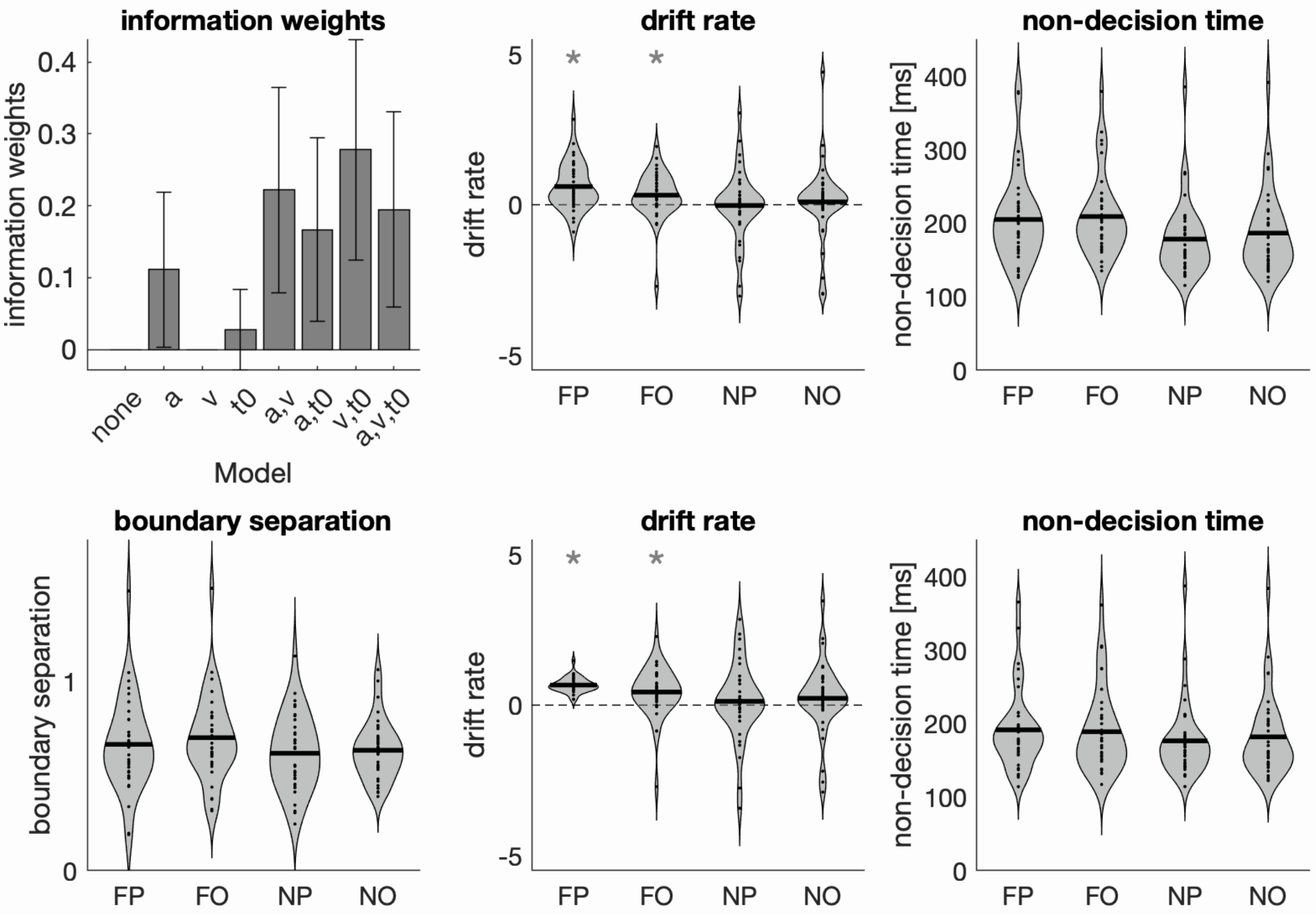

Supplementary Figure S3. Model selection and robustness of drift-diffusion parameters to inclusion of boundary separation parameter, related to Figure 5. Top left panel: Bar plot of information weights for the eight different model versions. Higher information weights denote more evidence for the respective model. Error bars denote the 95% confidence interval of between participant variability. The eight model versions comprise all possible combination of boundary separation, a, drift rate, v, and non-decision time, t0. Bottom row: Violin plot of drift-diffusion parameter for the model in which drift-rate, non-decision time and boundary separation was allowed to vary (a,v, t0). Drift rates (top center panel) and non-decision times (top right panel) of the best fitting model (v, t0) for comparison (same as Fig. 5). The statistical pattern of drift rates was not affected when the boundary separation parameter was allowed to vary (as judged by ANOVA effects). For the non-decision time, however, the main effect of inspection target (F > N) remains, yet the main effect of preview was not statistically significant. Instead, a main effect of preview (O > P) was found in the boundary separation parameter.

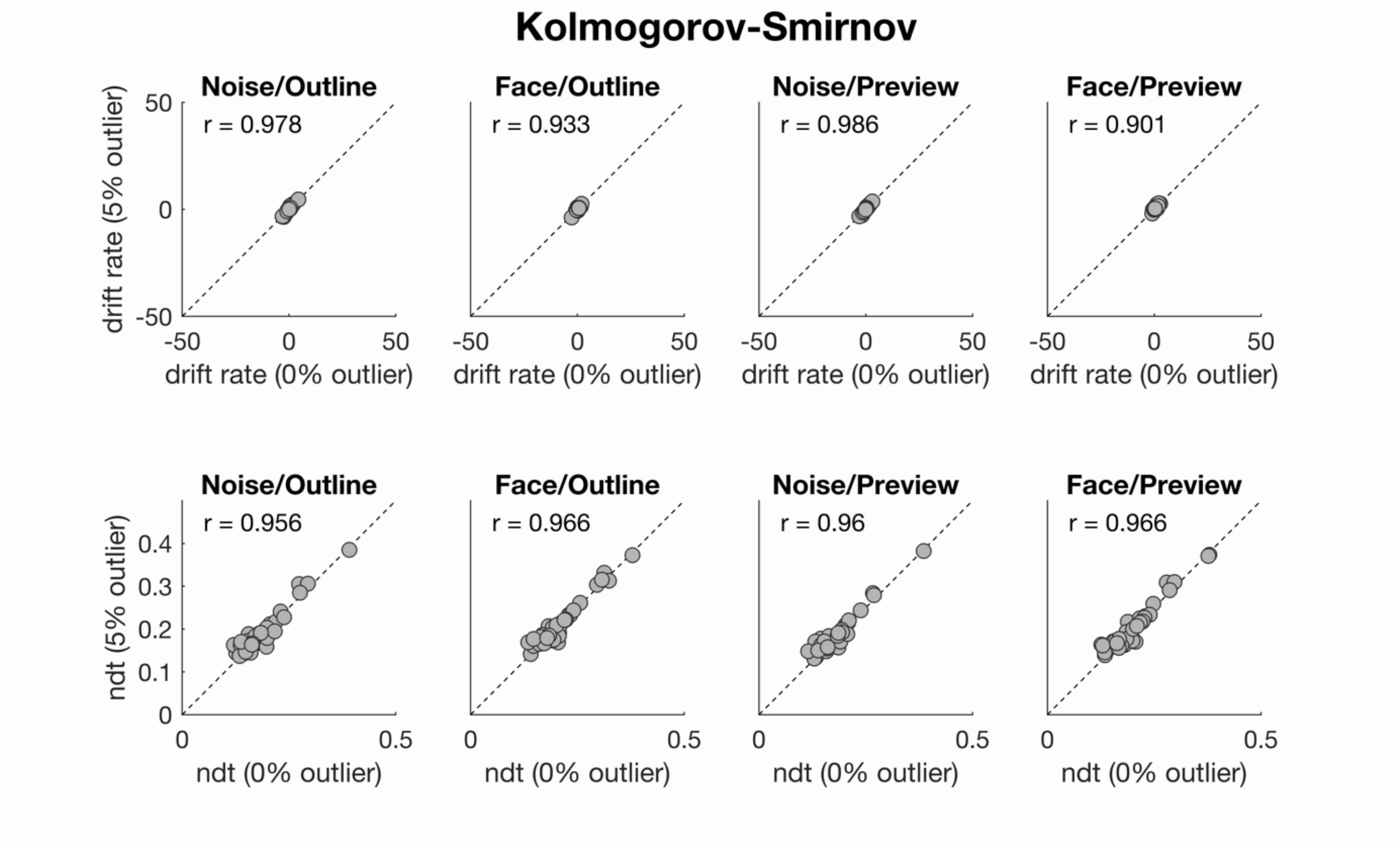

Supplementary Figure S4. Robustness of drift rates and non-decision times to outlier removal when using the Kolmogorov-Smirnov statistic as optimization criterion, related to Figure 5. The panels show the estimated drift rates (top row) non-decision times in seconds (ndt; bottom row) using the Kolmogorov-Smirnov statistic when 5% of outliers have been removed (ordinate axis) compared to when no outliers have been removed (abscissa) and the resulting Pearson correlation. Each data point denotes one individual and each panel in a row shows data from one of the four conditions.

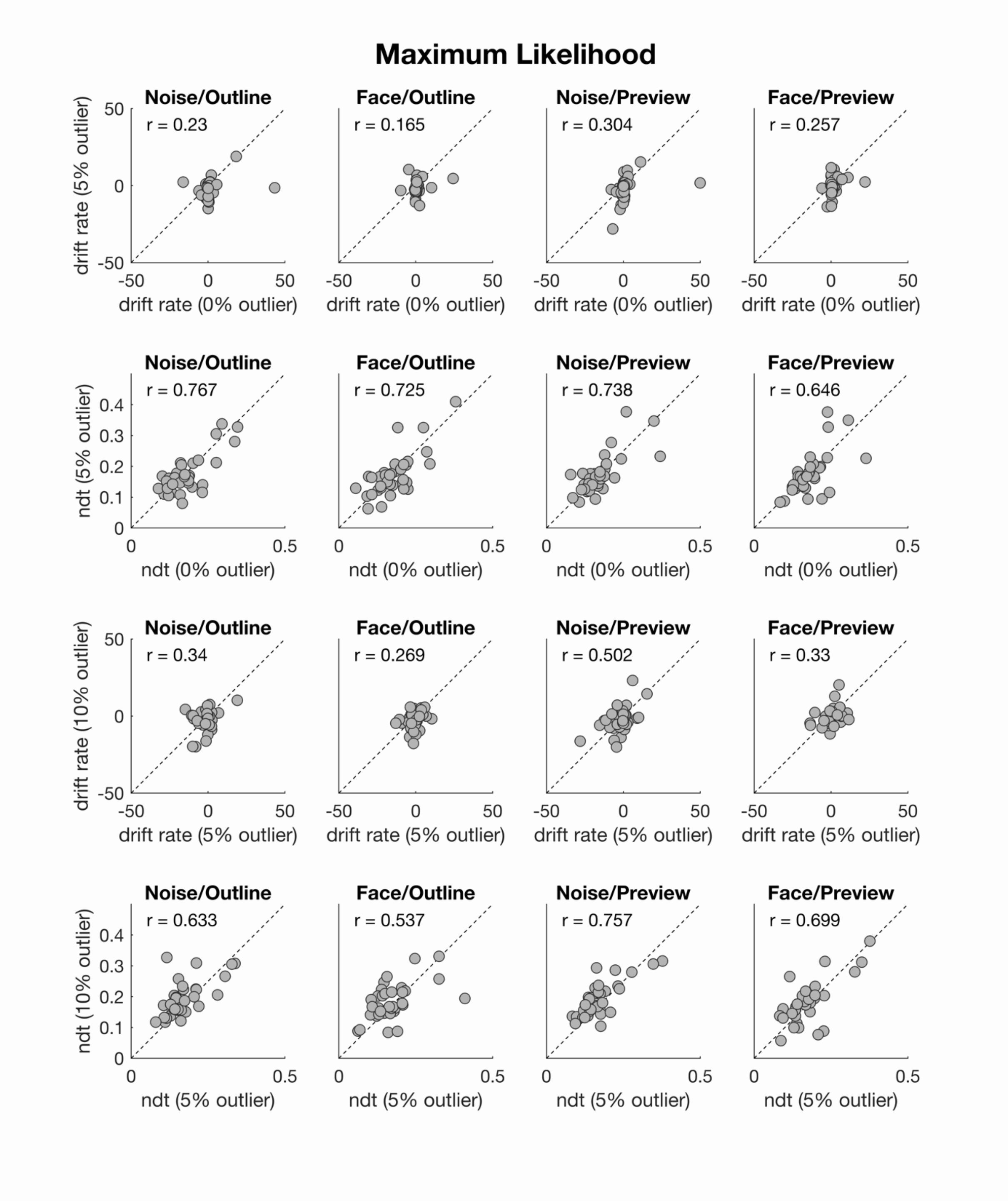

Supplementary Figure S5. Non-convergence of drift rates and non-decision times with increased outlier removal when using the maximum likelihood optimization criterion, related to Figure 5. Rows 1 and 2: Panels show the estimated drift rate (row 1) and non-decision time (ndt; row 2) using the maximum likelihood optimization criterion when 5% of outliers have been removed (ordinate axis) compared to when no outliers have been removed (abscissa). The two bottom rows show the same comparisons for 10% versus 5% of outlier removal. Each data point denotes one individual, and each panel with a row shows data from one of the four conditions. The resulting Pearson correlation is depicted in each panel.
